# Reproductive outcomes of different management strategies after hysteroscopic resection of the uterine septum with endometrial polyps: a retrospective study

**DOI:** 10.7717/peerj.20669

**Published:** 2026-01-28

**Authors:** Kaili Wang, Jianmin Du, Xinxin Zhao, Xin Zhao, Canyu Li

**Affiliations:** 1 Department of Obstetrics and Gynecology, The Third Affiliated Hospital of Zhengzhou University, Zhengzhou, China; 2 Department of Imaging, The Third Affiliated Hospital of Zhengzhou University, Zhengzhou, China; 3 Tianjian Laboratory of Advanced Biomedical Sciences, Institute of Advanced Biomedical Sciences, Zhengzhou, China

**Keywords:** Uterine septum, Endometrial polyps, Reproductive outcomes, Intrauterine adhesion, Placental abnormality

## Abstract

**Background:**

The presence of a septate uterus combined with endometrial polyps significantly impacts women’s fertility. There is currently no study on whether medication is needed after surgery and which postoperative regimen is more beneficial for uterine recovery and pregnancy outcomes. This study aims to compare the reproductive outcomes and complications of artificial cycle therapy with those of short-acting contraceptives or no hormonal treatment after hysteroscopic resection of the uterine septum with coexisting endometrial polyps.

**Methods:**

A retrospective study was conducted on 189 women with a history of infertility or adverse pregnancy who underwent hysteroscopic resection of uterine septum with endometrial polyps between December 2017 and February 2023 . According to the postoperative medication regimens, patients were divided into three groups: artificial cycle (Group A), short-acting contraceptive (Group B), and no hormonal treatment (Group C). The primary outcome was pregnancy rates leading to live birth within 12 months post-surgery.

**Results:**

There were 92 patients in Group A, 52 in Group B, and 45 in Group C. The live birth rates were 40.2% in Group A, 34.6% in Group B, and 31.1% in Group C (*χ*^2^ = 1.192, *P* = 0.547). A multivariate logistic regression analysis was performed incorporating confounding variables including age, body mass index (BMI), types of fertility problems, and types of uterine septum. The results showed that only age (adjusted odds ratio (OR) = 0.892, 95%CI [0.822–0.968], *P* = 0.006) was significantly associated with live birth after surgery. The mean time to pregnancy resulting in live birth was 9.6 months in Group A, 10.2 months in Group B, and 10.4 months in Group C (log-rank *P* = 0.468). There were no significant differences in clinical pregnancy rate, pregnancy loss rate, preterm birth rate, placental abnormality rate, postoperative intrauterine adhesion rate, and endometrial polyp recurrence rate among the three groups (*P* > 0.05).

**Conclusions:**

Hormonal therapy, including artificial cycles and short-acting contraceptives, may not be necessary after hysteroscopic septum resection with polypectomy for patients with short-term fertility requirements.

## Background

The uterine septum, a common congenital anomaly with an incidence of 2% to 3% in women of childbearing age (Rikken et al., 2019), is associated with recurrent pregnancy loss and obstetric complications (Prior et al., 2018; Practice Committee of the American Society for Reproductive Medicine, 2024; Turocy & Rackow, 2019). The guidelines from the Practice Committee of the American Society for Reproductive Medicine (ASRM) recommend surgical intervention for patients with a uterine septum and a history of recurrent pregnancy loss, following thorough consultation (Practice Committee of the American Society for Reproductive Medicine, 2024). The development of intrauterine adhesions is a major complication following septum resection, which can affect subsequent pregnancy outcomes and increase the risk of placental abnormalities (Practice Committee of the American Society for Reproductive Medicine, 2024; Shen et al., 2022b). The reported incidence of intrauterine adhesions varies greatly among different management protocols after septum resection, ranging from 0 to 37.5% (Tonguc et al., 2010; Shen et al., 2022a). Estrogen therapy is thought to promote endometrial repair and thereby prevent intrauterine adhesion (Ma et al., 2021). However, some early studies have suggested that endogenous estrogen is sufficient for endometrial regeneration and repair, making supplementation with exogenous estrogen unnecessary (Yu et al., 2016; Roy et al., 2014). The efficacy of such methods remains debated.

Patients with a uterine septum are sometimes accompanied by endometrial polyps. One study reported that the concurrent incidence of endometrial polyps in women with a uterine septum was approximately 25 to 33% (Xiong et al., 2024). Endometrial polyps can further impede fertility through mechanisms such as physical obstruction, the inflammatory response, and decreased endometrial receptivity (Vitale et al., 2021). Although approximately 25% of endometrial polyps, particularly those less than 1.0 cm in diameter, may resolve spontaneously (Bougie et al., 2024), performing endometrial polypectomy is recommended for women with fertility needs (Gu et al., 2018). Hysteroscopic surgery can ensure complete removal of the polyp and its base under direct visualization (Sheng & Lyons, 2020). The recurrence rates of endometrial polyps vary widely from 2.5% to 46% (Liu et al., 2021; Ciscato, Zare & Fadare, 2020). Estrogen receptors are highly expressed in the glandular tissue of endometrial polyps, leading to an excessive response of the endometrium to estrogen, which results in the formation of polyps. Consequently, progestins are frequently administered to inhibit the recurrence of polyps following surgical intervention (Raz et al., 2021; Tanos et al., 2017; Gündüz et al., 2022).

The artificial hormone cycle, consisting of sequential administration of estrogen and progestin, aims to facilitate the rapid regeneration of the endometrium at sites of injury within the uterine cavity, thereby preventing the formation of adhesions (Healy et al., 2016). Short-acting oral contraceptives contain low doses of estrogen and progestin, wherein the potent progestin component exerts a significant inhibitory effect on endometrial proliferation. As a result, these contraceptives are commonly utilized as a strategy to prevent the recurrence of polyps in women with recent fertility considerations (Raz et al., 2021). For patients with fertility needs after resection of the uterine septum combined with endometrial polyps, the optimal approach to restoring the uterine cavity while simultaneously preventing polyp recurrence remains undefined. This study aims to compare the outcomes of artificial hormone cycles, short-acting contraceptives, or no medication to establish evidence-based recommendations for postoperative management in this patient population.

### Methods

### Study population and Data collection

We performed a retrospective study including patients diagnosed with a uterine septum combined with endometrial polyps who underwent surgical treatment at The Third Affiliated Hospital of Zhengzhou University between December 2017 and February 2023. This study was approved by the Ethics Committee of The Third Affiliated Hospital of Zhengzhou University (Approval Number: 2023-271-01), and a waiver of informed consent was obtained. Postoperative data were collected through the outpatient system and telephone follow-up. The diagnosis of uterine septum was established according to the criteria set forth by the ASRM (Practice Committee of the American Society for Reproductive Medicine, 2024). In this study, the presence of a uterine septum in all patients was confirmed preoperatively through the use of transvaginal three-dimensional ultrasound imaging. The diagnosis of endometrial polyps was established through hysteroscopic surgery and subsequent histopathological analysis.

The inclusion criteria were as follows: (1) history indicating either infertility or adverse pregnancy outcomes before surgical intervention within the age bracket of 20–39 years, with no oral hormonal therapy contraindications; and (2) simultaneous execution of hysteroscopic resection of the septum and polyp. The exclusion criteria were as follows: (1) the length of the residual uterine septum after surgery exceeded one cm; (2) no intention of conceiving in the short term after surgery; (3) other uterine cavity pathologies affecting the uterine environment, such as submucosal myoma, intrauterine adhesions, or endometrial hyperplasia at the initial surgery; and (4) any other known cause of infertility or adverse pregnancy.

### Surgical method

All operations were performed by senior gynecological endoscopists in our center. All operations followed standardized procedures to ensure technical consistency.

Uterine septum resection: starting from the free end of the septum tissue, utilization of needle electrodes, alternating in a symmetric, horizontal cutting motion along the midline toward the septum base. For a complete uterine septum, a water-filled balloon was first placed in the lower uterine segment on the opposite side to help determine the location and orientation to begin resection of the uterine septum. The initial incision occurs through the septum’s lower segment above the cervix’s internal opening, followed by a directed incision extending toward the uterine fundus through the created opening.

Endometrial polyp resection: implementing bipolar ring electrodes, polyps were excised at the base, with consideration given to their respective locations, sizes, and quantities.

**Postoperative drug method:**

**Group A (artificial cycle)**: The protocol involves the combination of estradiol valerate tablets and progesterone capsules. Specifically, estradiol valerate tablets (1 mg, once a day) are administered continuously for 21 days, and during the final five days, progesterone capsules (100 mg, twice a day) are added. This regimen is followed for a total of two cycles.

**Group B (short-acting contraceptive):** Postoperative oral administration of drospirosterone and ethinylestradiol tablets (II) (3 mg; 0.02 mg), one tablet every day, 28 days for one cycle, a total of two cycles.

**Group C (no treatment):** No hormonal therapy was performed after surgery.

### Outcomes measures and definitions

The primary outcome was pregnancy resulting in a live birth within 12 months after surgery. Live birth was defined as the birth of a living fetus at a gestational age of more than 24 weeks. The secondary outcomes included clinical pregnancy, pregnancy loss, and preterm birth, all of which resulted from conception within 12 months after surgery. In women with a live birth, placental abnormalities, including placental abruption, placenta accrete, and placenta previa were assessed. Women were followed until their next conception (if within 12 months after surgery) if pregnancy loss occurred within 1 year or until 12 months after surgery otherwise.

In addition, after two menstrual cycles, a second-look hysteroscopy was provided to all patients, and the incidence of postoperative uterine adhesions and endometrial polyps was recorded. If intraoperative adhesions in the uterine cavity were found, they were separated during the procedure, and postoperative management was performed according to the protocol for intrauterine adhesions. If recurrent endometrial polyps were discovered during the surgery, they were excised, and patients were recommended to start trying for pregnancy after the operation. In both of these situations, the pregnancy outcome was still followed up according to the original group.

### Statistical analysis

SPSS 26.0 was utilized for all statistical analyses. The Shapiro–Wilk test was employed to assess the normality of the continuous variables. Measurement data complying with a normal distribution were expressed as mean (standard deviation), and one-way analysis of variance was adopted; otherwise, the data were expressed as median (interquartile range), with the Kruskal-Wallis H rank sum test applied. Categorical variables were presented as *n* (%) and analyzed using the chi-square test. If the chi-square test conditions were not met, Fisher’s exact probability test was used instead. We used the log-rank test to construct Kaplan–Meier curves to compare cumulative live birth rates according to treatment groups. Potential confounders, including age, BMI, types of fertility problems, and types of uterine septum. were adjusted using multivariable logistic regression models to estimate the independent effects of treatment regimens. Adjusted odds ratios (ORs) with 95% confidence intervals (CIs) were reported for primary and secondary outcomes. *P* < 0.05 was considered statistically significant.

## Results

A total of 189 cases were included and divided into three groups: Group A (artificial cycle, *n* = 92), Group B (short-acting contraceptive, *n* = 52), and Group C (no treatment, *n* = 45). The baseline characteristics were similar across the three groups (Table 1). As shown in the table, there were no significant differences in age, BMI, previous number of induced abortions, types of fertility problems, and types of uterine septum.

**Table 1:** Baseline characteristics of three groups. The table presents a comparative analysis of baseline characteristics among three distinct cohorts (Groups A, B, and C) across multiple variables, including age, body mass index (BMI), history of induced abortions, categories of fertility disorders, and classifications of uterine septum. Data are reported as means with standard deviations, medians with interquartile ranges, or frequencies with percentages, as appropriate. Intergroup differences were evaluated using suitable statistical methods, such as one-way analysis, the Kruskal-Wallis H rank sum test, or the chi-square test.

Variables	Group A (*n* = 92)	Group B (*n* = 52)	Group C (*n* = 45)	*F*/*H*/*χ*^2^	*p*-value
Age, years	28.6 (3.9)	29.5 (4.1)	27.9 (4.0)	2.095	0.126
BMI, kg/m^2^	22.6 (20.7, 25.6)	24.9 (21.6, 27.5)	23.0 (21.1, 26.1)	4.361	0.113
Previous number of induced abortions				5.278	0.4263
0	62 (67.4%)	25 (48.1%)	26 (57.8%)		
1	21 (22.8%)	19 (36.5%)	13 (28.9%)		
≥2	9 (9.8%)	8 (15.4%)	6 (13.3%)		
Types of fertility problems				5.297	0.072
Infertility	44 (47.8%)	18 (34.6%)	13 (28.9%)		
Adverse pregnancy	48 (52.2%)	34 (65.4%)	32 (71.1%)		
Types of uterine septum				1.402	0.497
Complete	34 (37.0%)	15 (28.8%)	13 (28.9%)		
Incomplete	58 (63.0%)	37 (71.2%)	32 (71.1%)		

**Notes.**

Data are presented as means (standard deviation), median (interquartile range), or *n*(%).

### Reproductive outcomes

Table 2 presents a comparative analysis of pregnancy outcomes across the three treatment groups. In Group A, 37 out of 92 women (40.2%) achieved a live birth, compared to 18 out of 52 women (34.6%) in Group B and 14 out of 45 women (31.1%) in Group C (*χ*^2^ = 1.192, *P* = 0.547). There was also no evidence of a difference in clinical pregnancy, pregnancy loss, or preterm birth rates.

**Table 2:** Comparison of different pregnancy outcomes across the three groups. This table compares the rates of primary and secondary pregnancy outcomes among the three groups (A, B, and C). The primary outcome is live birth, while secondary outcomes include clinical pregnancy, pregnancy loss, and preterm birth. Data are presented as the number of cases followed by the percentage within each group. Group comparisons were performed using the chi-square test.

Pregnancy outcomes	Group A (*n* = 92)	Group B (*n* = 52)	Group C (*n* = 45)	*χ* ^2^	*p*-value
Primary outcome					
Live birth	37 (40.2%)	18 (34.6%)	14 (31.1%)	1.192	0.547
Secondary outcomes					
Clinical pregnancy	44 (47.8%)	22 (42.3%)	18 (40.0%)	0.882	0.66
Pregnancy loss	7 (7.6%)	5 (9.6%)	4 (8.9%)	0.336	0.891
Preterm birth	2 (2.2%)	2 (3.8%)	1 (2.2%)	0.668	0.846

**Notes.**

Data are *n*(%).

Multivariate Logistic regression analysis was performed after including confounding factors such as age, BMI, types of fertility problems, and types of uterine septum. The results showed that only age (adjusted OR = 0.892, 95%CI [0.822−0.968], *P* = 0.006) was significantly associated with live birth after surgery (Table 3).

**Table 3:** Multivariate logistic regression analysis for live birth after surgery. This table presents the results of a multivariate logistic regression analysis performed to identify factors independently associated with the likelihood of achieving a live birth following surgery.

Variable	*P*-value	Adjusted OR	95%CI
Age	0.006	0.892	0.822 to 0.968
BMI	0.777	0.987	0.904 to 1.078
Types of fertility problems (*vs* Infertility)	0.876	1.055	0.541 to 2.057
Types of uterine septum (*vs* Complete)	0.311	0.696	0.345 to 1.404
Postoperative medication			
A *vs* C	0.195	1.689	0.765 to 3.730
B *vs* C	0.417	1.447	0.593 to 3.530

**Figure 1:**
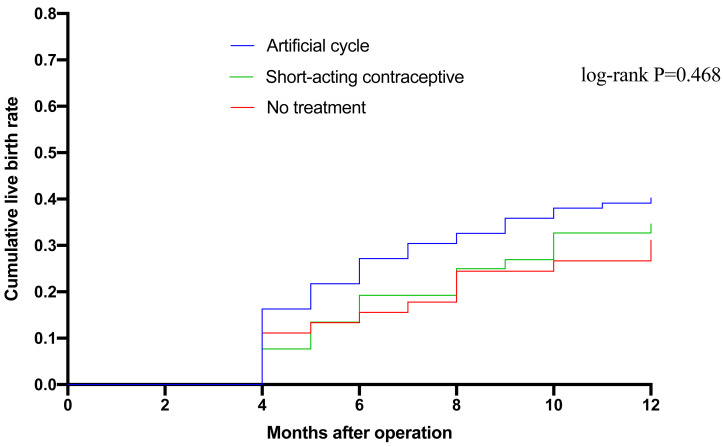
Kaplan–Meier analysis of the cumulative live birth rate in the three groups. Time is presented as months after surgery. The comparison of the survival curves was performed using the log-rank test, with the resulting *p*-value indicating no statistically significant difference in the cumulative live birth rates among the three intervention strategies over the observed period.

The mean time to pregnancy resulting in a live birth after surgery was 9.6 months (95%CI [8.9–10.3]) for Group A, 10.2 months (95%CI [9.4–11.0])) for Group B, and 10.4 months (95%CI [9.5–11.3]) for Group C (log-rank *P* = 0.468) (Fig. 1).

### Placental abnormalities

Table 4 compares placental abnormalities occurring during live birth among the three groups, showing an overall low incidence. Placenta accreta was always accompanied by placenta previa. Placental abruption occurred in 2 out of 37 women (5.4%) in Group A, with no cases in Groups B or C (*χ*^2^ = 1.077, *P* = 1.0). There was one case of placenta previa combined with placenta accreta in Group A and Group C, and one case of placenta previa alone in Group A and Group B, respectively. No significant differences were observed among groups (all *P* > 0.05).

**Table 4:** Comparison of placental abnormalities among the three groups. This table compares the incidence of specific placental abnormalities (abruption, accreta, and previa) across the three groups, focusing on the subpopulation of patients who achieved a live birth. Data are presented as the number and percentage of cases within each live birth cohort.

Placental abnormalities	Group A (*n* = 37)	Group B (*n* = 18)	Group C (*n* = 14)	*χ* ^2^	*p*-value
Placental abruption	2 (5.4%)	0	0	1.077	1.0
placenta accreta	1 (2.7%)	0	1 (7.1%)	1.580	0.432
Placenta previa	2 (5.4%)	1 (5.6%)	1 (7.1%)	0.511	1.0

**Notes.**

Data are *n*(%).

### Postoperative complications

Data on postoperative intrauterine adhesions and the recurrence of endometrial polyps are summarized in Table 5. Intrauterine adhesions were observed in 6 of 92 cases (6.5%) in Group A, 4 of 32 cases (7.7%) in Group B, and 2 of 45 cases (4.4%) in Group C, with no statistically significant differences between the groups (*χ*^2^ = 0.462, *P* = 0.863). Additionally, the recurrence rates of endometrial polyps observed in Group B was lower than Group A and Group C (1.9% *vs.* 7.6% *vs.* 11.1%), but the differences did not attain statistical significance (*χ*^2^ = 3.398, *P* = 0.182).

**Table 5:** Postoperative intrauterine adhesions and recurrence of endometrial polyps in the three groups. This table compares the incidence of two postoperative complications-intrauterine adhesions and recurrence of endometrial polyps-across the three treatment groups. Data are presented as the number and percentage of cases within each cohort.

Postoperative complications	Group A (*n* = 92)	Group B (*n* = 52)	Group C (*n* = 45)	*χ* ^2^	*P*-value
Intrauterine adhesions	6 (6.5%)	4 (7.7%)	2 (4.4%)	0.462	0.863
Endometrial polyps	7 (7.6%)	1 (1.9%)	5 (11.1%)	3.398	0.182

## Discussion

Our study showed that there were no significant differences in pregnancy outcomes and postoperative complications among the three groups of artificial cycle, short-acting contraceptives, and no medication. Despite expectations that hormonal therapy might reduce the risk of these complications, our results did not show any significant protective effect of either the artificial hormone cycle or short-acting contraceptives compared to no treatment.

It should be noted first that there is no consensus regarding the efficacy of uterine septum surgery in improving fertility outcomes (Rikken et al., 2020), despite the patient’s decision to proceed with the procedure for fertility purposes. According to the ASRM Practice Committee, surgical intervention is advised for patients presenting with a uterine septum accompanied by recurrent miscarriage. However, the current evidence substantiating a correlation between septum resection and improved infertility outcomes remains inadequate (Practice Committee of the American Society for Reproductive Medicine, 2024). Some studies showed that the excision of the uterine septum tissue, executed under hysteroscopic observation, endeavors to revitalize uterine cavity morphology and enhance pregnancy likelihood (Carrera et al., 2022; Tang et al., 2022). Noventa et al. (2022) concluded that uterine septum resection can reduce the rates of miscarriage and preterm birth. A randomized controlled trial conducted in 2021 compared hysteroscopic resection with observation management in patients with a septate uterus and found no significant difference in time to pregnancy or live birth rate (Rikken et al., 2021). Whether uterine septum surgery is necessary and whether surgery can improve pregnancy outcomes needs further study.

Previous studies have suggested that hysteroscopic procedures can increase the risk of placental abnormalities (Hajšek et al., 2022). Baldwin et al. (2018) noted that hysteroscopic interventions may lead to a higher likelihood of placenta accreta. The health of a pregnancy and proper placentation are influenced by the condition of the endometrium (Ng et al., 2020). A postoperative medication regimen for patients with a uterine septum combined with endometrial polyps aims to better restore the uterine environment, thereby enhancing the likelihood of successful pregnancy and reducing the rates of placental abnormalities. However, our findings suggested that the addition of hormonal therapy may not provide a clear advantage over expectant management without hormonal intervention. These results are in accordance with a previous prospective study on the effectiveness of estrogen following hysteroscopic septum resection, which found that estrogen therapy did not improve reproductive outcomes after the procedure (Roy et al., 2014). For patients with endometrial polyps, the use of progestins after surgery is to prevent the recurrence of polyps, and the guidelines suggest that patients with immediate fertility needs can attempt pregnancy without the need for postoperative medication (Bougie et al., 2024).

In our study, the incidence of intrauterine adhesions and polyps was low and comparable across all treatment groups, with no significant reduction in adhesion rates and polyps in the groups receiving hormonal therapy compared to those who did not. The rate of intrauterine adhesion in the three groups after the operation was 3.1−6.5%, which is consistent with the previously reported incidence of intrauterine adhesions after uterine septum surgery (Healy et al., 2016). Yu et al. (2016) conducted a cohort study on the incidence of intrauterine adhesions among patients who received estrogen, an intrauterine device, an intrauterine balloon, or no adjunctive treatment after uterine septum resection. Their findings indicated no significant difference in adhesion rates across these groups, implying that the uterine cavity wound was capable of complete spontaneous healing without the administration of supplementary estrogen therapy or other interventions (Yu et al., 2016). Short-acting contraceptives can promote endometrial growth through estrogen, helping to reduce the risk of intrauterine adhesion, while the strong and effective drospirenone can resist endometrial hyperplasia caused by the estrogen effect, resulting in its limited role in repairing wound endometrium (Healy et al., 2016). Regarding the risk of endometrial polyp recurrence, there is limited research on whether artificial cycles after polypectomy increase this risk (Raz et al., 2021; Tanos et al., 2017). The addition of a timely and appropriate dose of progesterone to the estrogen treatment regimen can inhibit the recurrence of endometrial polyps to a certain extent (Kossaï & Penault-Llorca, 2020). Our findings suggest that short-term, low-dose artificial cycle therapy does not increase the risk of postoperative polyps.

All patients included in this study were preoperatively diagnosed with a uterine septum by transvaginal three-dimensional ultrasound imaging, and the presence of endometrial polyps was subsequently confirmed through hysteroscopic surgery. While this approach ensured diagnostic accuracy for our cohort, we should acknowledge the inherent challenges in hysteroscopic diagnosis within clinical practice. A randomized controlled trial revealed that hysteroscopy, when used in isolation, is insufficient as a diagnostic tool for detecting a uterine septum. This limitation arises from its inability to adequately visualize the external contour of the uterus, thereby preventing definitive differentiation between a partial septate uterus and an arcuate uterus (Smit et al., 2015). This underscores the critical role of preoperative ultrasound in our diagnostic protocol (Practice Committee of the American Society for Reproductive Medicine, 2024). In addition, although hysteroscopic examination and hysteroscopic resection of endometrial polyps followed by pathological examination are the gold standards for diagnosing endometrial polyps (Sheng & Lyons, 2020), several studies have indicated that small endometrial polyps can be overlooked during hysteroscopic examination (Saponara et al., 2025; Campo et al., 2014), particularly in low-resource settings where hysteroscopists may have limited training or experience. Failure to detect them may lead to inadequate management of subfertility. This potential for oversight highlights the necessity of proper training and adherence to standardized diagnostic protocols to minimize missed diagnoses and ensure appropriate patient management.

This study has several limitations. As this was a retrospective study, a formal prospective sample size calculation was not performed. Given the sample size of 189 women, statistical analysis indicates that the study was sufficiently powered to detect differences exceeding 20% in live birth rates among the treatment strategies. However, the difference in live birth rates between the highest (Group A, 40.2%) and lowest (Group C, 31.1%) groups in our study was only 9.1%. Although no statistically significant difference in live birth rates was observed across the three groups, a post-hoc power analysis revealed that the sample size may have been insufficient to exclude a clinically meaningful difference. This limitation of statistical power is particularly evident in the case of our secondary outcomes, which exhibited very low event rates. The interpretation of our outcomes is limited by the risk of a Type II error. Our negative findings indicate that we did not find evidence for a large difference in postoperative therapy, rather than being able to draw equivalence conclusions in the different strategies. Moreover, the non-randomized assignment of treatments and the potential for unmeasured confounders (such as ovarian reserve and lifestyle) are inherent limitations of our retrospective design that may affect the robustness of the data. Furthermore, most patients began attempting pregnancy after a hysteroscopic follow-up at two months post-surgery if no abnormalities were found in the study, and did not undergo regular ultrasound examinations. As a result, it was not possible to assess the recurrence of endometrial polyps beyond two months postoperatively.

## Conclusions

The results of this study found no significant differences in reproductive outcomes and complication rates between the use of artificial cycles, short-acting contraceptives, or no hormonal therapy after uterine septum resection with concurrent polypectomy. However, due to the limited sample size and statistical power, these findings should be interpreted with caution. Therefore, clinicians can flexibly choose postoperative management plans based on individual conditions of patients (such as fertility plans, comorbidities, *etc*.), as there is no clear evidence from this study to support the recommendation of routine hormone therapy. This provides a preliminary basis for simplifying the postoperative management process and reducing medical costs. Future prospective studies with larger sample sizes are needed to confirm these findings and provide clearer guidance on the postoperative management strategy for this patient population.

##  Supplemental Information

10.7717/peerj.20669/supp-1 Supplemental Information 1 Raw data of patients with three postoperative management strategies for uterine septum with endometrial polyps in Excel format This is the primary dataset converted from SPSS into a universal Excel format. The first row of the spreadsheet contains the variable names with their value codes in parentheses.

10.7717/peerj.20669/supp-2 Supplemental Information 2 Description of the data Detailed documentation for the patient data including each variable within the dataset, specifying the variable name, its definition, classification (categorical or continuous), and the interpretation of all associated numerical codes.

10.7717/peerj.20669/supp-3 Supplemental Information 3 Patient data of three postoperative management strategies for uterine septum with endometrial polyps Use SPSS software to open this file.

## Additional Information and Declarations

### Data Availability

The following information was supplied regarding data availability: The raw data is available in the Supplemental Files.
