# Supplementary material for: Reproductive outcomes of different management strategies after hysteroscopic resection of the uterine septum with endometrial polyps: a retrospective study
Source: PeerJ. 2026 Jan 28;14:e20669. doi: 10.7717/peerj.20669 (PMC12860278; doi:10.7717/peerj.20669)
Supplement: Supplemental Information 2 — Detailed documentation for the patient data including each variable within the dataset, specifying the variable name, its definition, classification (categorical or continuous), and the interpretation of all associated numerical codes. [file peerj-14-20669-s002.docx]

Description of the data corresponding to the sav file

| variables | interpretation | types of variables | units/meaning of numerical codes |
| --- | --- | --- | --- |
| methods | Postoperative Management | categorical variable | 1 = "Artificial cycle";2 = "Short-acting contraceptive";3 = "No hormonal treatment" |
| BMI | Body mass index | continuous variable | kg/m^2^ |
| age | Age of patients | continuous variable | years |
| times | Previous number of induced abortions | continuous variable | times |
| types | Types of fertility problems | categorical variable | 1 = "Infertility";2 = "Adverse pregnancy" |
| septum | Types of uterine septum | categorical variable | 1 = "Complete";2 = "Incomplete" |
| polyps | Types of endometrial polyps | categorical variable | 1 = "Single";2 = "Multiple" |
| style | Types of uterine septum with endometrial polyps | categorical variable | 1 = "Incomplete with single";2 = "Incomplete with multiple";3 = "Complete with single";4 = "Complete with multiple" |
| end1 | Live birth | categorical variable | 0 = "No";1 = "Yes" |
| months | The months to pregnancy resulting in a live birth | continuous variable | months |
| end2 | Clinical pregnancy | categorical variable | 1 = "Not pregnant";2 = "Clinical pregnancy" |
| end3 | Pregnancy loss | categorical variable | 0 = "No";1 = "Yes" |
| end4 | Preterm birth | categorical variable | 0 = "No";1 = "Yes" |
| polypsR | Recurrence of endometrial polyps | categorical variable | 0 = "No";1 = "Recurrence" |
| adhesion | Intrauterine adhesion | categorical variable | 0 = "No";1 = "Intrauterine adhesion" |
| abruption | Placental abruption | categorical variable | 0 = "No";1 = "Yes" |
| accreta | Placenta accreta | categorical variable | 0 = "No";1 = "Yes" |
| previa | Placenta previa | categorical variable | 0 = "No";1 = "Yes" |
